# Measuring the Ocular Morphological Parameters of Guinea Pig Eye with Edge Detection and Curve Fitting

**DOI:** 10.1155/2020/6689023

**Published:** 2020-12-18

**Authors:** Yue Di, Ying Huang, Ya-jing Yang, Xing-Tao Zhou, Wen-ting Luo, Hai-yun Ye, Zhong-bao Qiao, Na Lu, Tong Qiao

**Affiliations:** ^1^Department of Ophthalmology, Shanghai Children's Hospital, Shanghai Jiaotong University, Shanghai 200062, China; ^2^Department of Ophthalmology, East Hospital Affiliated to Tongji University, Shanghai 200120, China; ^3^Department of Ophthalmology, Eye & ENT Hospital Fudan University, Shanghai 200031, China; ^4^Department of Radiology, Huashan Hospital North, Fudan University, Shanghai 200040, China

## Abstract

**Aim:**

To identify the guinea pig eyeball with edge detection and curve fitting and devise a noncontact technology of measuring ocular morphological parameters of small experimental animal.

**Methods:**

Thirty-nine eyeballs of guinea pig eyeballs were photographed to obtain the anterior and posterior surface; transverse and sagittal planes after the eyeballs were eviscerated. Next, the eyeball photos were input into digital image analysis software; the corresponding photo pixels-actual length ratio was acquired by a proportional scale. The contour lines of the eyeballs were identified by edge detection technology; conic curve fitting was applied to fit the contour line of the eyeball. The maximum and minimum diameters, the horizontal and vertical diameters, eccentricity, tilt angle, cross-sectional area, equatorial circumference, retrobulbar equatorial maximum length, corneal radius of curvature (CRC) in central region, and the whole cornea were calculated according to the geometric principles. The corneal data of in vitro study were compared with the in vivo results.

**Results:**

The contour line of the selected guinea pig eye was identified correctly by edge detection. There were no significant differences between anterior and posterior surfaces of one eyeball in the maximum diameters, eccentricity, cross-sectional area, equatorial circumference, and tilt angle (*P* > 0.01). There were significant differences of eccentricity and *CRC* between central region and the whole cornea (*P* < 0.01). There were no significant differences between keratometer in vivo and cornea in vitro.

**Conclusion:**

It was feasible to measure an experimental animal eye in a noncontact way. Edge detection and curve fitting technology could accurately evaluate the ocular morphological parameters.

## 1. Introduction

**Figure 1:**
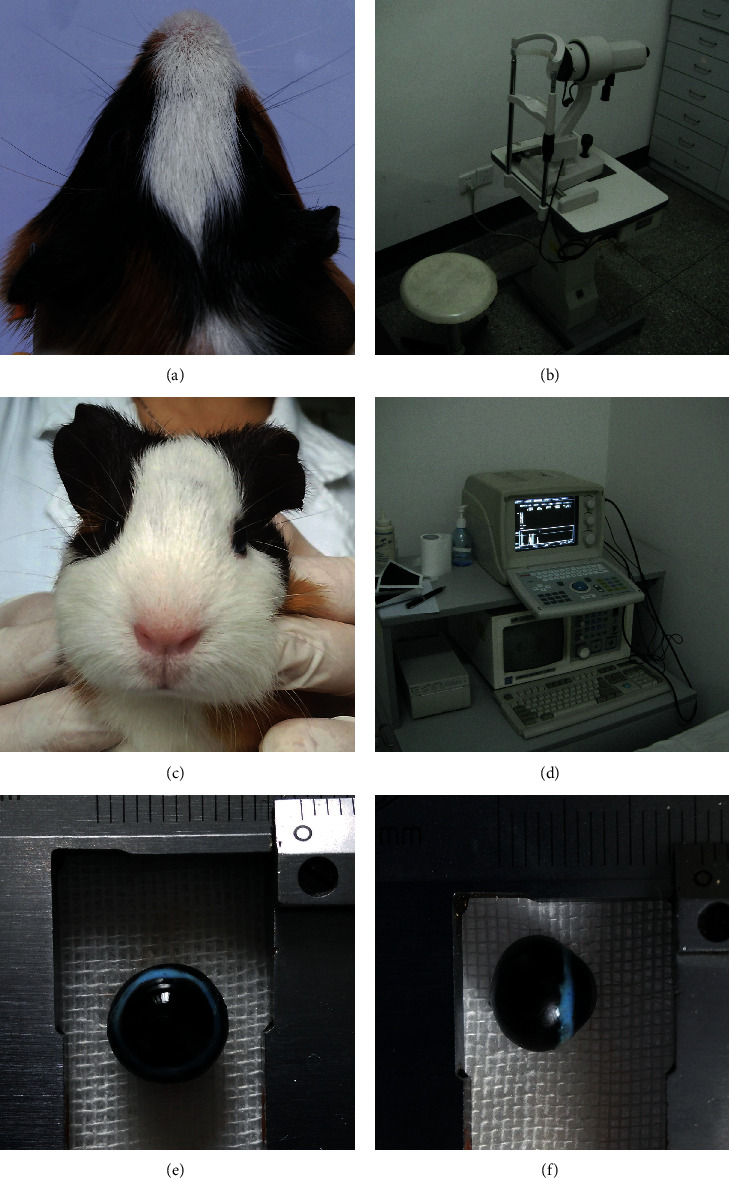
(a) The guinea pig eyeball is merely one-third the size of the human eye. (b) Keratometer (OM-4, Japan, Topcon), it is used to measure CRC. (c) The operator has to keep holding the conscious guinea pig throughout measurement. (d) A/B ultrasound (ODM-2100), which is used for axial length of small experimental animals currently. (e, f) The coronal and sagittal plane of the eyeball in vitro. The guinea pig eyes were very soft; it is easy to deform. Thus, it was impossible to measure with calipers directly.

Guinea pigs have been introduced as a promising animal model in recent years [1–3]. They are cooperative, inexpensive, and easy to handle. Thus, the guinea pigs have been regarded as an appropriate object for the study of environmental factors involved in refraction development [3–5]. However, the ocular morphological data measurement processed in small animals like guinea pigs is still a difficult task. The guinea pig eye is merely one-third the size of the human eye, and the corneal radius of curvature (CRC) is much steeper and out of the range of conventional keratometry (Figure 1(a)). Because of this, Norton and McBrien [5–7] proposed to measure the CRC with a plus 8.0 D aspherical lens. A set of stainless steel ball-bearings was used for calibration (Figure 1(b)). Nevertheless, the guinea pig was poorly cooperated during measuring, which inevitably led to unreliable results (Figure 1(c)). Similarly, the axial length measurement of guinea pig usually involved topical anesthesia due to the high fatality rate of general anesthetics [8, 9]. Such a measurement in the waking state required higher skills for the manipulator. The ultrasound probe could not reliably be set completely perpendicular to the corneal center. The eyeball would be compressed during contact measurement (Figure 1(d)). All these factors impacted the accuracy of the final result.

Given the difficulty of measuring in vivo, a better choice was to eviscerate the eyeball and assess a fully exposed eyeball statically. Nevertheless, the eyeball in vitro was very soft and easy to be compressed if any contact measurement was applied (Figure 1(e)). Moreover, the eyeball shape in coronal plane was similar to a tilt ellipse, and the symmetry axis was difficult to determine (Figure 1(f)). Thus, some valuable morphological parameters, such as eccentricity, cross-sectional area, equatorial circumference, and the maximum and minimum diameters, would be unavailable. Therefore, it was very necessary to develop a noncontact measuring method with digital image processing technology.

In this respect, matrix laboratory, as a numerical computing environment and programming language [10–12], produces two-dimensional graphics and supports developing applications with graphical user interface features. Edge detection and curve fitting technologies are both significant mathematical methods in the areas of computer vision [13, 14]. The former identifies points in a digital image at which the image brightness changes sharply [15], while the latter is in the process of constructing a curve which has the best fit to a series of data points [16, 17]. Curve fitting technology can determine the best visual fit of circular or elliptical arcs, such as the contour of an eyeball, and then transformed the edge information into a curve equation [18, 19]. In doing so, a mathematical model is developed, with a series of geometrical principles are utilized.

Our current study designed a series of methods to identify the image of the guinea pig eye with digital image processing technology and proposed a noncontact approach to measure ocular morphological parameters without artificial factors. Our work is presented as follows.

## 2. Animals and Biometric Measurements In Vivo

Twenty-one guinea pigs (English short hair stock, tricolor strain, three weeks of age) were obtained from the laboratory of Fudan University. Totally, there are thirty-nine eyeballs (right: 18, left: 21). No corneal diseases were observed in a slit lamp. This current study was carried out in strict accordance with the recommendations in the Guide for the Care and Use of Laboratory Animals of the National Institutes of Health. Our work has been approved by the Institutional Animal Care and Use Committee. All surgery was performed under sodium pentobarbital anesthesia, and all efforts were made to minimize suffering.

The radius of corneal curvature (CRC) was measured in alert guinea pigs with a keratometer (OM-4; Topcon, Tokyo, Japan) combined with a plus 8.0 D aspherical lens. A set of stainless steel ball-bearings was used for calibration. The CRC for each animal was measured in triplicate (Figure 1(b)).

## 3. Numerical Simulation Techniques of the Eyeball Photo In Vitro

### 3.1. Method of Actual Length-Photo Pixels Conversion

**Figure 2:**
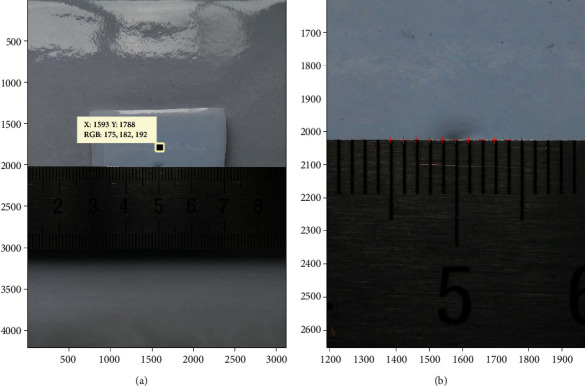
(a) After the photo was input into graphics, any point in this photo had a definite coordinate in a two-dimensional coordinate system (*x*-3120, *y*-4280). As shown in the illustration, the coordinate of the selected point was 1593 and 1788. (b) The corresponding pixel of an actual 1 mm distance in the center was *L*_1mm_ = (*L*_1_ + *L*_2_ + *L*_3_+⋯.+*L*_10_)/10 = 39.502 pixels, namely, the ratio-coefficient was 0.0253 (1/39.502); the distance between any two points could be calculated.

A 13-megapixel digital camera (Macro Mode) was fixed with a 10 cm brace over a platform with a pure white background. The length × width of the photos was 3120 × 4280 pixels. After the selected photos were input into the MATLAB software, they were equivalent to a two-dimensional coordinate system with *x*-coordinates 0-3120 and *y*-coordinates 0-4280 (Figure 2(a)). We get the access to the MATLAB license which is authorized by Shanghai Jiaotong University.

First, one calibrated scale was placed horizontally in the center of the platform and elevated by 5 mm, which was equivalent to the height of the guinea pig eye center. The calibrated scale photo was input into two-dimensional graphics. Two points (*m*_1_, *n*_1_) and (*m*_2,_*n*_2_), which were 1 mm apart in the calibrated scale center, were selected with the *getpts* function. According to the principle of geometry, the distance of any two points, (*m*_1_, *n*_1_) and (*m*_2,_*n*_2_), in the coordinate system could be computed (formula: $L_{mn}=\sqrt{{\left(m_{1}-m_{2}\right)}^{2}+{\left(n_{1}-n_{2}\right)}^{2}}$). In this way, the corresponding pixels of actual 1 mm distance in the center could be available.

To improve the accuracy of the conversion ratio, ten contiguous 1 mm distances,*L*_1_, *L*_2_, *L*_3_.⋯*L*_10_, in the center of the calibrated scale were selected. The average number of pixels was calculated. Thus, the corresponding pixels of actual 1 mm distance in the center were *L*_1mm_ = (*L*_1_ + *L*_2_ + *L*_3_+⋯.+*L*_10_)/10. In doing so, the distance between any two points in the center of the photo could be converted to the actual distance by this ratio-coefficient (Figure 2(b)). To verify the accuracy of this coefficient, 10 mm, 4 mm, and 12 mm actual lengths in the center were randomly selected, and the computed results were compared.

Because all the photos were obtained from one camera and saved as the same size, the position was fixed. Therefore, the ratio-coefficient (0.0253) was also applicable to all the following eyeball photos.

### 3.2. Acquisition of Ocular Edge Data

**Figure 3:**
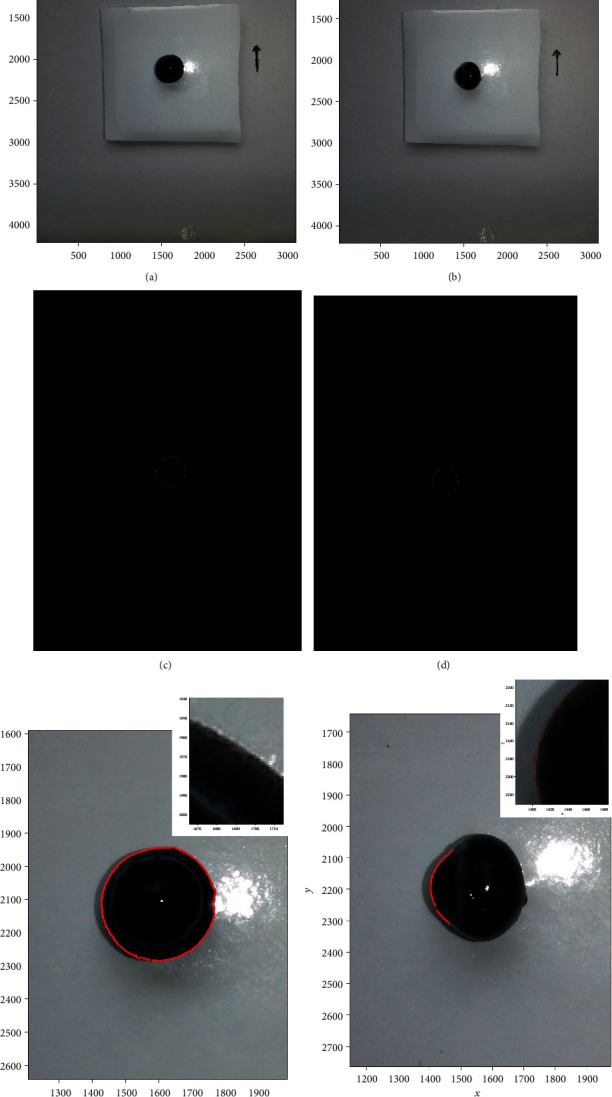
(a, b) The eyeball of the selected guinea pig was placed cornea upside (a) and cornea left (b) on the center of the platform. A point was marked by a glowing needle tip at the top of the corneoscleral limbus for location (red arrow). The eyeball was photographed to obtain the coronal and transverse views in this way. The white-black ocular images in the coronal (c) and transverse views (d). (e, f) The points at which image brightness changed sharply were organized into a set of curved line segments termed edges (red points).

First, the guinea pig was sacrificed. A point was marked by a glowing needle tip at the top of the corneoscleral limbus before eviscerating the eyeball. Next, the eyeball was removed, and the bulbar conjunctiva was excised. Afterward, the eyeball was placed in the center of the platform and shoot with a camera from four directions: anterior and posterior surface and transverse and sagittal plane. The center of the platform was slightly pitted for fastening. Canny edge detection algorithm was applied to obtain the dual-threshold value image. The points at which the image brightness changed sharply were organized into a set of curved line segments termed edges (Figures 3(e) and 3(f)). Further, the nontarget edge was removed, and merely, the eyeball edge was reserved. The images were saved; then, the *find* function was used to acquire the coordinate data of the eyeball edge including coronal and horizontal views in the 3120 × 4280 coordinate system. The illustration is shown in Figure 3; only the parts of coronal and transverse plane were presented for convenience.

### 3.3. Mathematical Simulation: Conic Equation Fitting

**Figure 4:**
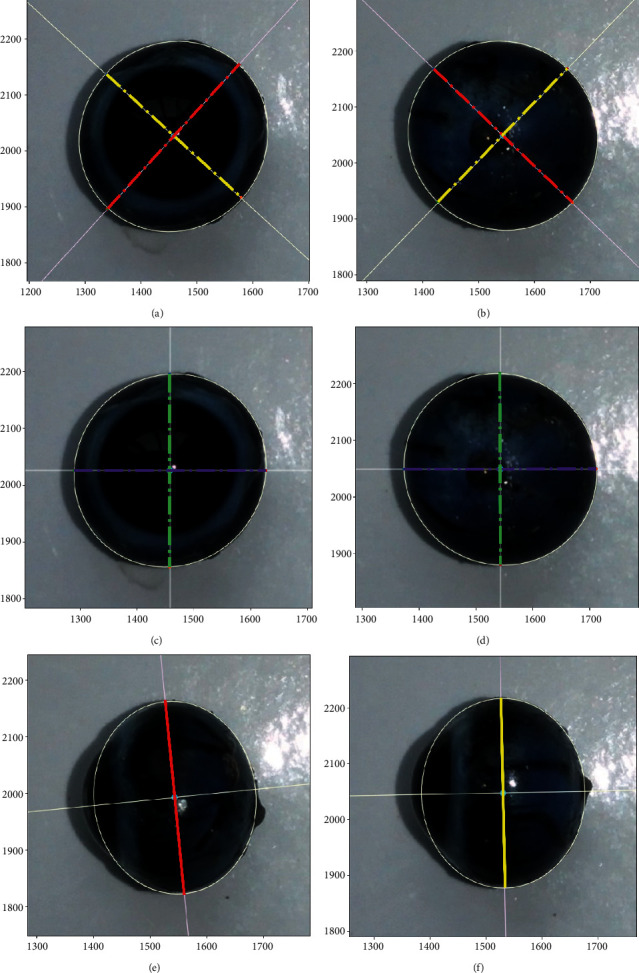
(a, b) The coronal plane. Conic curve fitting on anterior surface and posterior surface of eyeball. (c, d) The coronal plane. (e, f) Results of retrobulbar pole portion conic curve fitting on transverse plane and sagittal plane. Note: the red dashed line: maximum diameters; yellow dashed line: minimum diameters. The purple dashed line: horizontal diameters; the green dashed line: vertical diameters. The solid line: retrobulbar equatorial maximum length.

The eyeball edge in the coronal view includes anterior and posterior surface was like a tilt ellipse curve. Such a curve was quite suitable to fit with the conic equation (*Ax*^2^ + *Bxy* + *Cy*^2^ + *Dx* + *Ey* + *F* = 0). As shown in Figures 4(a) and 4(b), the eyeball edge was converted to a binary quadratic equation after the conic curve fitting.

#### 3.3.1. Calculation of Tilt Angle and Central Point

According to the principle of geometry, the central point (Xc, Yc) of the conic equation (*Ax*^2^ + *Bxy* + *Cy*^2^ + *Dx* + *Ey* + *F* = 0) could be calculated:

(1) $$\begin{array}{c} \text{Xc}=\frac{BE-2CD}{4AC-B2},\\ \text{Yc}=\frac{BD-2AE}{4AC-B2}. \end{array}$$

The formula of the tilt angle was:

(2) $$\begin{array}{c} \text{Angle}=\arctan\left(\frac{B}{\left(A-C\right)}\right)/2, \end{array}$$

In such a way, the central point and the tilt angle of the eyeball in the coronal view could be calculated (Figures 4(a) and 4(b)).

#### 3.3.2. Calculation of the Maximum and Minimum Diameters

Given the tilt angle of an ellipse, the slope of the major axis was *k*_aa_ = tan(90 ± angle), and the slope of the minor axis was *k*_bb_ = −1/*k*_*aa*_.

Given the central point (Xc, Yc) and the two slopes of the axis (*k*_aa_, *k*_bb_), the line equations (*y* = *kx* + *b*) of the two axes were both available (Figure 4(b), formula: *y* = *k*_aa_ × *x* + *b*_aa_, *y* = *k*_bb_ × *x* + *b*_bb_).

Given the linear equations of the two axes and the ellipse equation, we solved the binary quadric equations with the *solve* function:

(3) $$\begin{array}{c} \text{Formula}:\left\{\begin{array}{c} y=kx+b,\\ {Ax}^{2}+Bxy+{Cy}^{2}+Dx+Ey+F=0. \end{array}\right. \end{array}$$

The results were two points (*x*_1_, *y*_1_) and (*x*_2_, *y*_2_) which were the intersections of the symmetry axis and ellipse. The pixel distance between (*x*_1_, *y*_1_) and (*x*_2_, *y*_2_) was:

(4) $$\begin{array}{c} \text{Formula}:\;L=\sqrt{{\left(x^{1}-x_{2}\right)}^{2}+{\left(y_{1}-y_{2}\right)}^{2}}. \end{array}$$

In this way, the line lengths within the ellipse, namely, the maximum and minimum diameters (*L*_aa_, *L*_bb_), of the eyeball in the coronal view were obtained. After the actual length-photo pixel conversion, the actual lengths of the maximum and minimum diameters were both available (formula: *L*_actual_ = *L*_aa_(or *L*_bb_)/*L*_1mm_).

#### 3.3.3. Calculation of Eccentricity, Cross-Sectional Area, and Equatorial Circumference

Given the length of the maximum and minimum diameters, the eccentricity could be available (formula: $e=\sqrt{{\left(L_{\text{aa}}/2\right)}^{2}-{\left(L_{\text{bb}}/2\right)}^{2}}/L_{\text{aa}}$).

The equatorial circumference (C) was:

(5) $$\begin{array}{c} C=2\times \pi \times \left(\frac{L_{\text{bb}}}{L_{1\text{mm}}}\right)+4\times \left(\left(\frac{L_{\text{aa}}}{L_{1\text{mm}}}\right)-\left(\frac{L_{\text{bb}}}{L_{1\text{mm}}}\right)\right). \end{array}$$

The cross-sectional area (S) was:

(6) $$\begin{array}{c} \text{Formula}:S=\pi \times a\times b=3.1416\times \left(\frac{L_{\text{aa}}}{L_{1\text{mm}}}\right)\times \left(\frac{L_{\text{bb}}}{L_{1\text{mm}}}\right). \end{array}$$

#### 3.3.4. Calculation of the Horizontal and Vertical Diameters

Because the horizontal and vertical lines both passed through the center of the ellipse (Xc,Yc), we can calculate the horizontal and vertical diameters. The system of binary quadric equations was solved with the *solve* function.

(7) $$\begin{array}{c} \text{Formula}:\left\{\begin{array}{c} y=\text{Yc},\\ {Ax}^{2}+Bxy+{Cy}^{2}+Dx+Ey+F=0. \end{array}\right. \end{array}$$

The results were two points which were the intersections of the horizontal line and ellipse. Similarly, the equations:

(8) $$\begin{array}{c} \text{Formula}:\left\{\begin{array}{c} x=\text{Xc},\\ {Ax}^{2}+Bxy+{Cy}^{2}+Dx+Ey+F=0. \end{array}\right. \end{array}$$

The results were the intersections of the horizontal or vertical line with ellipse (Figures 4(c) and 4(d)).

The distance between (*x*_1_, *y*_1_) and (*x*_2_, *y*_2_) was also computed by the formula:

(9) $$\begin{array}{c} L=\sqrt{{\left(x^{1}-x_{2}\right)}^{2}+{\left(y_{1}-y_{2}\right)}^{2}}. \end{array}$$

After the actual length-photo pixel conversion, the length of horizontal and vertical diameters could be calculated.

Through above methods, the morphological parameters of the eyeball were obtained. To assess the accuracy of this measurement, paired *t*-test was chosen to compare the maximum and minimum diameters, eccentricity, horizontal diameter, vertical diameter, cross-sectional area, and equatorial circumference of the anterior and posterior surface of eyeball. *P* < 0.01 was a significant difference (Table 1).

### 3.4. Calculation of Eccentricity, CRC, and Corneal Curvature

According to the definition of CRC, the central 3 mm region of the cornea was similar to a circle whose radius was the CRC. However, a guinea pig's eyeball is only one-third the size of the human eye. To evaluate the impact of corneal aspherical feature, the conic equation (*Ax*^2^ + *Bxy* + *Cy*^2^ + *Dx* + *Ey* + *F* = 0) was applied first to fit the central region of the cornea (3 mm) and the whole cornea (6 mm) in transverse and sagittal plane. The eccentricity can be obtained from the fitted ellipse. In the present study, the *nlinfit* and *round* function were applied to the circle to fit the central region and the whole cornea. These two fitting curves were compared; the radius of the fitting circle was the CRC. Then, the actual CRC and corneal curvature could be calculated (Formula: *R*_actual_ = *R* × 1/*L*_1mm_).

## 4. Results

### 4.1. Reliability Assessment of Actual Length-Photo Pixel Conversion

**Figure 5:**
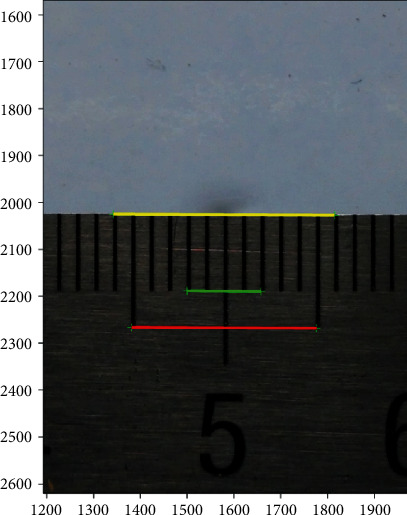
For example, the pixel distance between two green points in this photo, (a1, b1) and (a2, b2), was $L_{ab}=\sqrt{{\left(a_{1}-a_{2}\right)}^{2}+{\left(b_{1}-b_{2}\right)}^{2}}$. The pixel distance of the red line in this photo was *L*_*ab*_ = 395.6016 pixels. The pixel distance of the green line in this photo was *L*_*ab*_ = 158.0064 pixels. The pixel distance of the red line in this photo was *L*_*ab*_ = 395.6016 pixels. The pixel distance of the yellow line in this photo was *L*_*ab*_ = 473.2102 pixels. After the conversion, the actual length of the red line was *L*_actual_ = *L*_*ab*_ × 1/*L*_1mm_ = *L*_*ab*_ × 1/39.502 = 10.0142 mm. The actual length of the green line was *L*_actual_ = *L*_*ab*_ × 1/*L*_1mm_ = *L*_*ab*_ × 1/39.502 = 4.0000 mm. The actual length of the yellow line was *L*_actual_ = *L*_*ab*_ × 1/*L*_1mm_ = *L*_*ab*_ × 1/39.502 = 11.9794 mm.

The corresponding pixels of the actual 1 mm distance in the center were *L*_1mm_ = (*L*_1_ + *L*_2_ + *L*_3_+⋯.+*L*_10_)/10 = 39.502 pixels. As shown in Figure 5, the actual length of the red line was *L*_actual_ = *L*_*ab*_ × 1/*L*_1mm_ = *L*_*ab*_ × 1/39.502 = 10.0142 mm. The actual length of the green line was *L*_actual_ = *L*_*ab*_ × 1/*L*_1mm_ = *L*_*ab*_ × 1/39.502 = 4.0000 mm. The actual length of the yellow line was *L*_actual_ = *L*_*ab*_ × 1/*L*_1mm_ = *L*_*ab*_ × 1/39.502 = 11.9794 mm.

### 4.2. Comparison of Ocular Morphological Parameters between Anterior and Posterior Surface of the Eyeball

The results of ocular morphological parameters between anterior and posterior surface of the eyeball are shown in Table 1. There were no significant differences between anterior and posterior surface of one eyeball in the maximum and minimum diameters, eccentricity, cross-sectional area, and equatorial circumference (paired *t*-test, *P* > 0.01). Similarly, there was no statistically significant difference between the angle_long axis_ of anterior surface and 180°- angle_long axis_ of posterior surface (*P* > 0.01).

**Figure 6:**
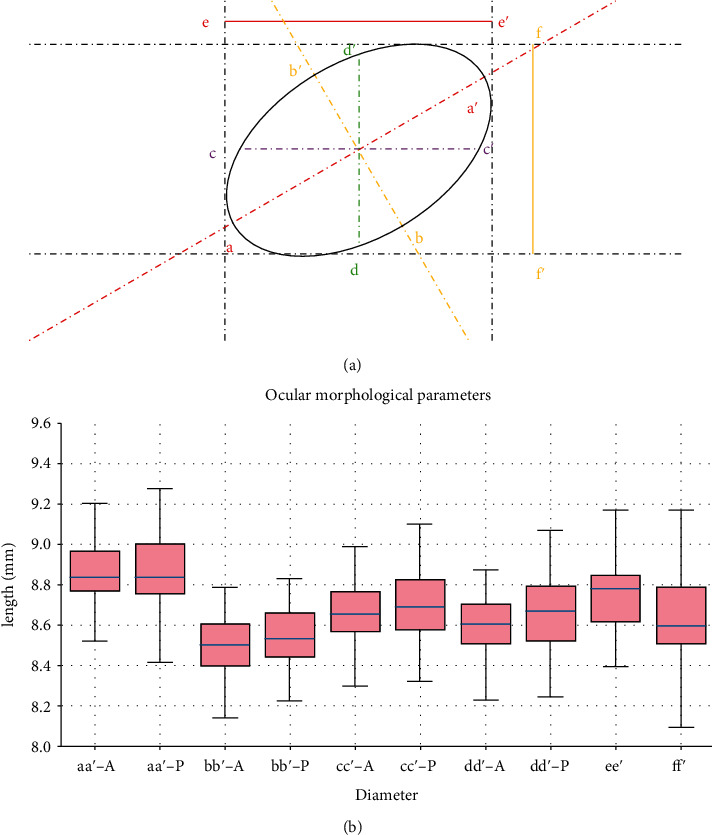
(a) To simulate the contour of eyeball with a simplified graph: aa′ and bb′: maximum and minimum diameters; cc′ and dd′: vertical and horizontal diameters; ee′ and ff′ retrobulbar equatorial maximum length in transverse plane and sagittal plane. (b) -A was diameters on anterior surface; -P was on posterior surface.

The comparison of ocular morphological diameters is shown in Figure 6.

### 4.3. Comparison of Eccentricity and CRC in Different Regions of the Cornea

**Figure 7:**
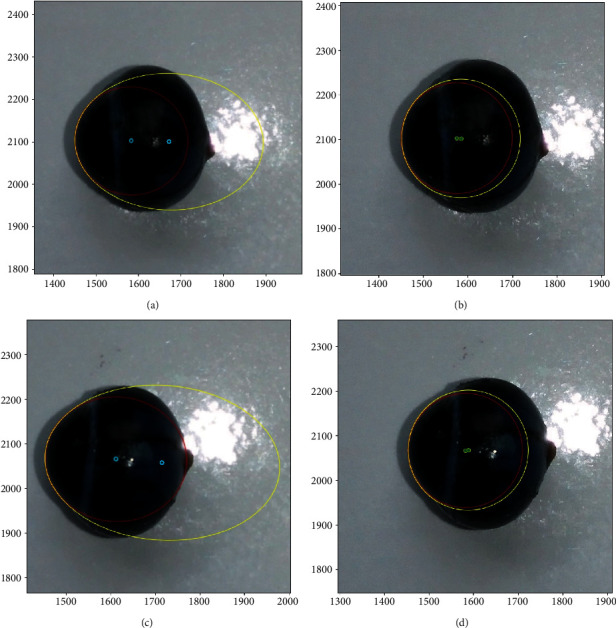
(a, b) The transverse plane of the eyeball. (c, d) The sagittal plane of the eyeball. Conic fitting and circle fitting between the central cornea (red line) and the whole cornea (yellow line).

As shown in Figure 7(a), after conic curve fitting, the whole cornea was similar to an ellipse. Meanwhile, the central region of the cornea was more like a circle. The eccentricity, CRC, and corneal curvature were compared; significant differences were found between central cornea and whole cornea region (paired *t*-test, *P* < 0.01). The results are shown in Table 2.

### 4.4. Comparison of Corneal Curvature In Vivo with Keratometer and CRC In Vitro with Numerical Simulation

**Figure 8:**
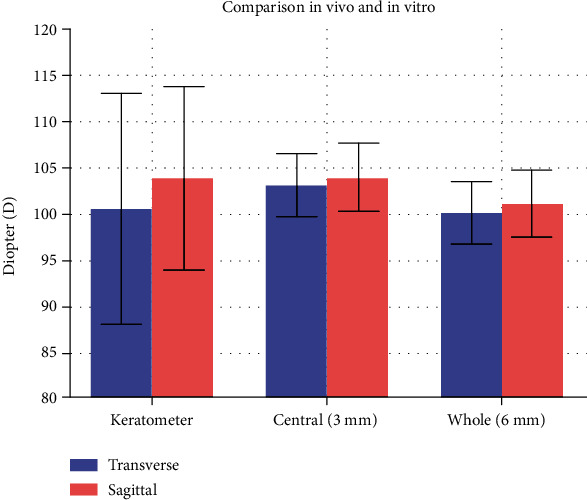
Comparison of corneal curvature in vivo with keratometer and CRC in vitro with numerical simulation. There was no significant difference between keratometer in vivo and cornea in vitro (paired *t*-test, *P* > 0.05).

The comparison of corneal curvature in vivo with keratometer and corneal curvature in vitro with numerical simulation is shown in Figure 8. The corneal curvature in vivo with keratometer in the transverse and sagittal plane was 100.5 ± 12.5 D and 103.8 ± 9.9 D, respectively. There was no significant difference between keratometer in vivo and cornea in vitro (paired *t*-test, *P* > 0.05).

Transverse planes are as follows:

Keratometer in vivo vs. central cornea in vitro: paired *t*-test, *P* = 0.19

Keratometer in vivo vs. whole cornea in vitro: paired *t*-test, *P* = 0.82

Sagittal planes are as follows:

Keratometer in vivo vs. central cornea in vitro: paired *t*-test, *P* = 0.94

Keratometer in vivo vs. whole cornea in vitro: paired *t*-test, *P* = 0.07

## 5. Discussion

### 5.1. Scale Conversion Ratio of Photo Pixels and the Corresponding Actual Length

The present study proved it was feasible to identify and digitally measure an experimental animal eye using a noncontact approach. As shown in Figure 5, the lengths in a real scale were randomly selected. After a ratio conversion, the distances of two points in the photo were 10.0142 mm (approximately 10 mm), 4.0000 mm (approximately 4 mm), and 11.9794 mm (approximately 12 mm). Therefore, the conversion result was believable. To measure the eyeball as accurately as possible, the resolution of the photos needed to be sufficiently high. It was even more important to obtain the scale conversion ratio of the photo pixels and the corresponding actual length as correctly as possible. To achieve this, it was necessary to place a proportional scale in the photo. However, if the scale was placed beside the eyeball, the image might be deformed outside of the central region because of the very close object distance. Moreover, if every eyeball photo was required to calculate the scale conversion ratio, the computing process would be inefficient, and the results would be inevitably influenced in the end. To resolve this problem, the current method fixed the resolution ratio of the photo and made sure the shooting position was fixed. In this way, the scale conversion ratio was obtained by placing the proportional scale in the center and the ratio applied to the subsequent eyeball photos. Therefore, the efficiency was significantly improved. Furthermore, our study selected ten 1 mm distances in the center of the photo and calculated the average number of pixels. The scale ruler was elevated by 5 mm high, which was equivalent to the center height of the guinea pig eye. All these designs helped to ensure the reliability of measurement results.

### 5.2. Edge Detection Technology in Ocular Morphological Measurement

Edge detection comprises a set of mathematical approaches that aimed at identifying points in a digital image at which the image brightness changed strikingly [20]. Edge detection had been widely applied in various computer vision systems, as it is an important technique to extract useful structural information from different vision objects and dramatically reduced the amount of data processed [15, 16, 21]. As shown in Figure 3(a)–3(b), the eyeball was placed on a white background. After edge detection, the points at which image brightness changed sharply were organized into a set of curved line segments termed edges (Figures 3(c) and 3(d)). Then, the nontarget edge was removed, and the edge of eyeball could be identified accurately. There are multiple edge detection algorithms including “Sobel”, “Canny”, “Prewitt”, “Roberts”, and “Log” [15]. The Canny edge detector was developed by John F. Canny in 1986 [22]. It uses a multistage algorithm to detect a wide range of edges in images. Evidently, the clarity of the picture will obviously affect the accuracy of the measurement. Besides, too much illumination can lead to shadows, and the edge will be blurry instead. Thus, a disperse illumination is recommended.

### 5.3. Curve Fitting Method with the Least Squares

In the present study, the contour edge of the eyeball in the coronal plane was a tilt ellipse. In mathematics, the conic section (syntax: *Ax*^2^ + *Bxy* + *Cy*^2^ + *Dx* + *Ey* + *F* = 0) is a curve obtained with the intersection of a cone with a plane. Conic sections have certain spherical properties that make them a meaningful expansion set for the description of general arc curves such as corneal surfaces in the fields of optical engineering and physiological optics [23]. According to the principle of geometry, the central point, the tilt angle, and the maximum and minimum diameters of the eyeball in the coronal view could be calculated. Therefore, some valuable parameters, such as eccentricity, cross-sectional area, and equatorial circumference, which could not be acquired in the past, could be analyzed.

In the current study, curve fitting technique was proved to be a practical method to fit the contour edge of guinea pig's eyeball. After edge detection, the contour edge of the eyeball was merely consisted of a series of adjacent data points (Figures 3(e) and 3(f)), which could not be analyzed directly [24]. In the field of data visualization, least square is a method of fitting a curve to data points to minimize the sum of the squares of the distances of the points from the curve [25]. Curve fitting constructs an optimal fitting curve based on the least squares [26]. On the coronal plane, the morphological parameters between the anterior and posterior surface of the eyeball were compared; no significant differences were found between the maximum diameter, cross-sectional area, equatorial circumference, and horizontal and vertical diameters. Nevertheless, the difference between the minimum diameter and the eccentricity was statistically significant, but the standard deviation of the data set was small. The reason may be related to the protrusion of residual optic disc on posterior surface. Besides, the tilt angles of the maximum diameter between the anterior surface and posterior surface were complementary, indicating the eyeballs in the two photos were mirror-symmetric. The results were satisfactory overall.

### 5.4. Calculation of the CRC with Mathematical Simulation

According to the definition of the radius of the circle (CRC) in the human eyes, the central 3 mm region of the cornea is similar to a circle [27]. Thus, circle fitting was used to fit the surface of the cornea after edge detection, and then, the actual CRC could be calculated by ratio-coefficient. Because the eyeball was already taken out and placed in a horizontal position, the data of the CRC were more credible compared with the data measured by a keratometer in vivo. The standard deviation of the data measured by keratometer was proved more obvious than standard deviation of mathematical simulation. Meanwhile, the CRC from the whole cornea was larger than the CRC from the central region of the cornea (Figures 7(b) and 7(d)). The cornea of the guinea pig was aspheric, which was the same as the cornea of the human.

In mathematics, the eccentricity, abbreviated as *e*, is a useful indicator associated with every conic section. It is a measure of how much the conic section deviates from being circular. The eccentricity of a circle is zero; the eccentricity of an ellipse that is not a circle is greater than zero but less than 1. Corneal eccentricity is regarded as a helpful indicator in the diagnosis of keratoconus in humans [28]. In the present study, conic fitting was used to fit the central region and the whole cornea. Significant differences were found between central cornea and whole cornea region. Apparently, the cornea of a guinea pig was similar to the cornea of a human. The central region is close to a circle, while the peripheral regions are relatively smooth.

The shortcoming of this method was it focused on the eyeball in vitro; thus, the changes in the eyeball could not be observed through a longitudinal study. In addition, the computational process was relatively complex. Nevertheless, such a method could offset the lack of instrument and software which aimed at small animal eyeballs in experimental research. This method used a noncontact measuring technology, and there were fewer artificial factors. The numerical computing and implementation of algorithms in a numerical computing environment could determine some valuable parameters, such as the eccentricity, equatorial circumference and cross-sectional area, and the maximum and minimum diameters. Therefore, this digital image processing technology is more accurate and credible compared to conventional measurements.

## Figures and Tables

**Table 1:** Comparison of ocular morphological parameters between anterior and posterior surface.

Parameters	Anterior surface	Posterior surface	*t*	*P*
Maximum axis (mm)	8.87 ± 0.17	8.86 ± 0.19	0.30	0.77
Minimum axis (mm)	8.49 ± 0.15	8.53 ± 0.16	4.15	<0.01
Eccentricity	0.29 ± 0.04	0.27 ± 0.04	3.41	<0.01
Horizontal axis (mm)	8.68 ± 0.16	8.69 ± 0.19	-0.70	0.48
Vertical axis (mm)	8.61 ± 0.20	8.65 ± 0.19	-2.31	0.03
Transverse area (mm^2^)	59.09 ± 2.06	59.35 ± 2.22	-1.93	0.06
Perimeter (mm)	27.41 ± 0.48	27.45 ± 0.53	-1.93	0.06
Tilt angle (°)	84.82 ± 55.13	92.20 ± 55.74	-1.15	0.26

Note: the tilt angle in the table in anterior surface was angle_long axis_; in contrast, the tilt angle in posterior surface was 180°- angle_long axis_.

**Table 2:** Comparison of eccentricity, CRC, and corneal curvature between central cornea and whole cornea.

Plane	Cornea	Eccentricity	CRC	Corneal curvature
Transverse	Central (3 mm)	0.55 ± 0.15	3.28 ± 0.11 mm	102.97 ± 3.32 D
	Whole (6 mm)	0.67 ± 0.10	3.38 ± 0.12 mm	100.00 ± 3.38 D
	*t*	-4.87	-6.62	6.77
	*P*	<0.01	<0.01	<0.01
Sagittal	Central (3 mm)	0.54 ± 0.17	3.25 ± 0.11 mm	103.93 ± 3.65 D
	Whole (6 mm)	0.64 ± 0.14	3.35 ± 0.12 mm	100.91 ± 3.64 D
	*t*	-4.79	-6.65	6.44
	*P*	<0.01	<0.01	<0.01

## Data Availability

The data used to support the findings of this study are available from the corresponding author upon request.
